# Mr. Bampfield on Bleeding in Hydrophobia

**Published:** 1812-12

**Authors:** R. W. Bampfield

**Affiliations:** H. M. S. Warrior, Downs


### *To the Editors of the Medical and Physical Journal.*

GENTLEMEN,

IN the last Number of your Journal, Dr. Kinglake, in his valuable speculations on Hydrophobia, has, with illustrating acumen, traced the strong resemblance that subsists between the real character of that disease and that of Gastritis. Many of the symptoms of Hydrophobia enumerated, strictly correspond with the definition of Gastritis in Cullen's Nosology; the appearances, on dissection, satisfactorily evince the previous existence of inflammation of the stomach and œsophagus in that disease, and afford clear traces of an identity of inflammatory character. From general reasoning, and the particular analogy which obtains between the inflammatory symptoms, and the appearances, on dissection, of Hydrophobia and Gastritis, Dr. K. has drawn a rational deduction, that the mode of treatment, universally sanctioned and adopted in Gastritis, is indicated in and applicable to, Hydrophobia; but he does not offer any cases or facts illustrative of the efficacy of venesection and the system of depletion.

It has been my study to deposit in the archives of my memory, every remarkable case that has been drawn within the scope of my knowledge or the circle of my reading, that exhibits a successful mode of treating those diseases, which have been considered the opprobrium of the profession, and have resisted the suggestions of ingenious reasoning, or have defeated the tests of experiment. On the subject of Hydrophobia, my memory supplies me with two cases cured by copious bleeding: one is recorded in an old Magazine. A countryman of Norfolk, in a paroxysm of Hydrophobia, cut himself, and bled so profusely, before medical assistance could be procured, that his death was immediately apprehended. He, however, recovered both from Hydrophobia and the debility consequent on the great loss of blood. Another case brought to a successful issue by bleeding, is recorded in an American Journal, by (I believe) Dr. Burton. These facts have been long strongly impressed on my mind; and determined me to employ profuse bleeding, (even to endanger life, already in jeopardy, if necessary,) should I ever have to conduct the treatment of an hydrophobist, and they render Dr. K.'s plan of cure not only "feasible," but strictly legal and judicious.*

The poison of a rabid animal is not singular in exercising its malignant influence on the stomach; the poison of the snakes cobra di capello, and cobra di manille, in India, produces an opposite effect from excitement, for it quickly affects the stomach with deadly torpor and peculiar pain, that require the use of the strongest stimulants, and the nervous and circulating systems are apparently influenced by sympathy with the stomach.

I have seen one case of Hydrophobia, at the London Hospital, treated unsuccessfully by continual mercurial friction. At the same hospital, many recent bites of dogs reputed and believed to be rabid, were treated with invariable success by excision, and dressing the wounds with ung. hydrargyri fort. until slight ptyalism was induced.

Might not the experiment of abstracting copious quantities of blood be tried on rabid animals with great chance of success, and the result admitted as a test of its utility and efficacy?

A reason for not having attained a successful method of treating this tremendous disorder, may be probably found in the rarity of its occurrence: few medical men possess an opportunity of conducting the treatment of it more than once or twice during their lives; and but few, in their first essays on a disease that is new to them, are bold and hazard-

---

* The following case, extracted from a recent newspaper, is in point; but we know not the authority on which it stands.—Editors.

*Hydrophobia.*—On Tuesday, the 5th of May, A. Bheestie, who had been bitten three weeks before in the leg by a mad dog, was carried to the Native Hospital, about three o'clock in the afternoon, with the symptoms of hydrophobia strongly upon him. He was immediately bled to the extent of forty ounces. The symptoms of the disease yielded in succession as the blood flowed; and, before the vein was closed, he stretched out his hand for a cup of water, and calmly drank it off, though the mere approach of the water but a few minutes before had thrown him into convulsions. After the bleeding, he lay down on a cot, fell asleep, and continued so for nearly two hours. When he awoke, the symptoms of the disease were threatening to return; another vein was then opened, and eight ounces more of blood were taken away, which so completely subdued the disease, that he has had not a symptom of it since.—*Kilkenny Chronicle.*

ous ous enough to incur the blame of departing from the doctrines of their teachers, or the practice (however unsuccessful) they have witnessed. Might it not be useful to send every case of Hydrophobia that occurs in the country to the county hospital, and in the metropolis to some centrical one, where the modes of treatment may be varied; their result recorded; and the experience of ages be brought into one guiding view, that would direct to further experiment?

*H. M. S. Warrior,* R. W. BAMPFIELD.

*Downs, October* 12, 1812.

